# Mathematical Modeling Predicts That Strict Social Distancing Measures Would Be Needed to Shorten the Duration of Waves of COVID-19 Infections in Vietnam

**DOI:** 10.3389/fpubh.2020.559693

**Published:** 2021-01-12

**Authors:** Anass Bouchnita, Abdennasser Chekroun, Aissam Jebrane

**Affiliations:** ^1^Equipe Systèmes Complexes et Interactions, Ecole Centrale Casablanca, Casablanca, Morocco; ^2^Laboratoire d'Analyse Nonlinéaire et Mathématiques Appliquées, University of Tlemcen, Tlemcen, Algeria

**Keywords:** COVID-19, SARS-CoV-2, epidemic model, multi-scale modeling, basic reproduction number

## Abstract

Coronavirus disease 2019 (COVID-19) emerged in Wuhan, China in 2019, has spread throughout the world and has since then been declared a pandemic. As a result, COVID-19 has caused a major threat to global public health. In this paper, we use mathematical modeling to analyze the reported data of COVID-19 cases in Vietnam and study the impact of non-pharmaceutical interventions. To achieve this, two models are used to describe the transmission dynamics of COVID-19. The first model belongs to the susceptible-exposed-infectious-recovered (SEIR) type and is used to compute the basic reproduction number. The second model adopts a multi-scale approach which explicitly integrates the movement of each individual. Numerical simulations are conducted to quantify the effects of social distancing measures on the spread of COVID-19 in urban areas of Vietnam. Both models show that the adoption of relaxed social distancing measures reduces the number of infected cases but does not shorten the duration of the epidemic waves. Whereas, more strict measures would lead to the containment of each epidemic wave in one and a half months.

## 1. Introduction

The coronavirus disease 2019 (COVID-19) emerged in Wuhan, China and has caused a pandemic that has affected almost all countries around the globe. In the absence of effective vaccines or therapeutics against COVID-19, countries have resorted to non-pharmaceutical interventions (NPIs) to slow down the spread of the epidemic. These interventions have been proven to be effective against epidemics, such as SARS and 2009 swine flu, however, it is not clear what effects they have on the spread of COVID-19 as it is a novel disease. Mathematical modeling offers the opportunity to quantify the impact of these interventions and to design effective strategies that contain the spread of COVID-19.

Vietnam has been affected by the pandemic, but it has managed to control the epidemic in comparison with other countries. The first COVID-19 case in Vietnam was reported on Janruary 23, 2020. As of April 12, 2020, 260 positive SARS-CoV-2 tests were confirmed, and no deaths were reported. These results indicate that Vietnam adopted one of the most effective epidemic control strategies in the world. Indeed, the country was well-prepared to slow down the spread of COVID-19 due to its experience in containing the SARS epidemic. The non-pharmaceutical interventions that have been adopted by Vietnam include preventing people from visiting areas with an elevated risk of infection, social distancing, school closures, shutting down borders with China, and isolating infected individuals. A nationwide lockdown was mandated from 1 to 15 April 2020. It consisted of limiting the movement of individuals to reduce the chances of disease transmission.

In this work, two mathematical models are used to analyze the dynamics of the spread of COVID-19 in Vietnam. The first is an SEIR model in which the population is divided into four compartments: susceptible, exposed, infectious, and recovered. We derive an analytical estimate for the basic reproduction number using this model and calculate it for the case of Vietnam. After model calibration using the reported data, we used it to study the impact of different levels of social distancing measures on the spread of the disease. The second model uses a multi-scale architecture to explicitly describe the transmission dynamics at the level of individuals (1, 2). The movement of individuals is simulated using a social-force model. This model is studied to investigate the impact of limited movement of the population on the spread of the epidemic.

The remainder of the paper is organized as follows: section 2 presents a summary of related works and how the paper contributes to the research field. Section 3 introduces the two models and the data that is used to calibrate them. Section 4 presents the results of numerical simulations quantifying the impact of relaxed and strict social distancing measures on the spread of the disease. The contributions and the limitations of the study are discussed in section 5.

## 2. Related Works

To gain a better understanding of the COVID-19 spread in Vietnam, it is urgent to build mathematical models that consider the impact of non-pharmaceutical interventions. Compartmental models that are widely used to describe the transmission dynamics of infectious diseases can be used to study the evolution of the COVID-19 epidemic. These models usually consist of a system of ordinary differential equations (ODEs). A compartmental model was used to evaluate the impact of non-pharmaceutical interventions in China (3). A susceptible-infected-recovered (SIR) model was previously formulated to study the effect of quarantine in containing the spread of COVID-19 (4). Susceptible-exposed-infectious-recovered (SEIR) models are used to simulate the transmission dynamics of infectious diseases with a relatively long incubation period (5). A sensitivity analysis of an SEIR model for COVID-19 was presented in a previous study (6). This analysis has shown that the early detection, early isolation, early treatment, and a comprehensive treatment strategy are necessary for slowing down the spread of the disease. SIR and SEIR models can be used to calculate the basic reproduction number ($R_{0}$) (7, 8). The advantage of using the ODEs to implement compartmental models is the possibility to study the obtained systems analytically and numerically.

Individual-based modeling is another framework which can be used to implement compartmental models. Agent-based models explicitly describe the person-to-person transmission of the disease (9, 10). Some of these models describe the movement of individuals in space (11). Other individual-based models do not describe the movement of individuals but include several details on the spread of the disease under realistic conditions (12, 13). Multi-scale models integrate the processes regulating disease transmission at both the within-host and between-host levels (14). In this context, we have recently developed a multi-scale model to describe COVID-19 transmission dynamics in Italy, China, and Morocco (1, 2).

The main contribution of this work is that both continuous and agent-based approaches are used and compared to study the transmission dynamics of COVID-19 in Vietnam. We use two models to evaluate the impact of non-pharmaceutical interventions on the spread of the disease. The first belongs to the SEIR class and can be used to determine the basic reproduction number ($R_{0}$) while the second is a multi-scale model which implicitly captures the movement of individuals. The aim of this study is not to provide an accurate forecast of the evolution of the pandemic, but rather to gain a deeper understanding of the impact of non-pharmaceutical interventions on the spread of the disease. Both models quantify the impact of lockdown policies on the propagation of the COVID-19 pandemic in Vietnam.

## 3. Mathematical Modeling of COVID-19 Transmission Dynamics in Vietnam

### 3.1. Data Collection

The data on COVID-19 cases in Vietnam were collected from Ministry of Health (15). It shows that the number of daily cases has increased steadily over February and March. Then it started decreasing after the adoption of the social distancing measures on 31 March, 2020. Indeed, the Vietnamese government mandated a nationwide lockdown of 15 days from April 1, 2020 to April 15, 2020. During this lockdown, people are required to stay at home and not go out except for buying necessary goods or for going to work at factories. We have represented the collected data which include the daily and cumulative numbers of reported COVID-19 cases before and after the lockdown in Tables 1, 2, respectively.

**Table 1:** Number of daily and cumulative reported number of COVID-19 in Vietnam from March 6, 2020 to March 30, 2020 before the nationwide lockdown.

**Date (day/month)**	**Number of newly reported cases**	**Cumulative number**
6 March	1	17
7 March	4	21
8 March	9	30
9 March	1	31
10 March	3	34
11 March	4	38
12 March	6	44
13 March	3	47
14 March	6	53
15 March	5	58
16 March	3	61
17 March	5	66
18 March	2	68
19 March	8	76
20 March	11	87
21 March	5	92
22 March	14	106
23 March	15	121
24 March	2	123
25 March	11	134
26 March	19	153
27 March	10	163
28 March	11	174
29 March	14	188
30 March	15	203

**Table 2:** Number of daily and cumulative reported number of COVID-19 in Vietnam from March 31, 2020 to May 3, 2020 following the application of the lockdown.

**Date (day/month)**	**Number of newly reported cases**	**Cumulative number**
31 March	4	207
1 April	11	218
2 April	9	227
3 April	10	237
4 April	3	240
5 April	1	241
6 April	4	245
7 April	4	249
8 April	2	251
9 April	4	255
10 April	2	257
11 April	1	258
12 April	2	260
13 April	5	265
14 April	1	266
15 April	1	267
16 April	1	268
24 April	2	270
3 May	1	271

### 3.2. The SEIR Compartmental Model

**Figure 1:**
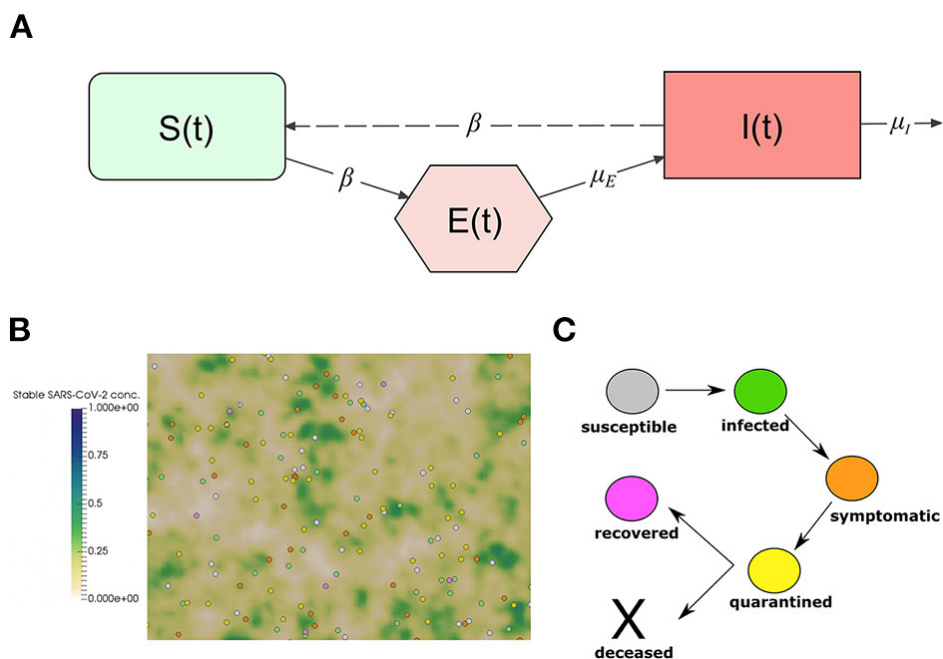
**(A)** Interactions between the compartments of the epidemiological model (1). The continuous lines represent transition between compartments, and entrance and exit of individuals. The dashed line represents the transmission of the infection through the interaction between susceptible and infected individuals. **(B)** Snapshot of a numerical simulation of the model. Spheres represent individual people moving in a square section of 250 ×250 m. The color of each individual represents the class to which it belongs: white for susceptible, green for infected, orange for symptomatic, yellow for quarantined, and pink for recovered. The concentration of stable SARS-CoV-2 on surfaces is represented using the gradient of the green color. **(C)** The clinical course of COVID-19 patients in the model.

We adapt the well-known SEIR model to describe the transmission dynamics of COVID-19 in Vietnam. The model splits the total population *N* in three classes: susceptible (*S*), exposed (*E*), and infected individuals (*I*). A schematic representation of the considered compartments and the interactions between them is represented in Figure 1A. It is an extension of the classical SIR Kermack and McKendrick model 1927 which includes a compartment of exposed individuals. We do not consider mortality in the model. This allows us to decouple *R* from the rest of the model. The model equations are, for *t* > 0,

(1) $$\begin{array}{l} \{\begin{array}{lll} S^{\prime }\left(t\right) & = & -\beta S\left(t\right)I\left(t\right),\\ E^{\prime }\left(t\right) & = & \beta S\left(t\right)I\left(t\right)-\mu_{E}E\left(t\right),\\ I^{\prime }\left(t\right) & = & -\mu_{I}I\left(t\right)+\mu_{E}E\left(t\right), \end{array} \end{array}$$

with the initial conditions

(2) $$\begin{array}{l} S\left(0\right)=S_{0},E\left(0\right)=E_{0},\text{and}I\left(0\right)=I_{0}. \end{array}$$

We consider that *E*_0_ = *R*_0_ = 0 (initially, there is no exposed and recovered individual). Moreover, we assume that *S*(0)+*E*(0)+*I*(0)+*R*(0) = 1 from which we have *S*(*t*)+*E*(*t*)+*I*(*t*)+*R*(*t*) = 1 for all *t* > 0. This means that we will work with the proportion to the total population. All the parameters of the model are described in Supplementary Table 1. Note that there is a slight difference in the used notations between our work and the study by Kuniya et al. (5). The identification parameter is denoted by ε in our paper, while it is denoted by *p* in the work by Kuniya et al. (5). Also, we refer to the onset rate by μ_*e*_ while in Kuniya et al. 2020, it is denoted by ε.

Inspired by a previous work (5), we consider an identification function

$$t\in {\mathbb{R}}^{+}\mapsto X\left(t\right)=\varepsilon \times I\left(t\right)\times N.$$

This quantity describes the number of infective individuals who are identified at time *t*, with *N* is the total population in Vietnam (*N* = 97, 338, 579) and ε is the identification rate. We can suppose that ε ∈ [0.01, 0.1] (5). It allows to take in consideration the uncertainty due to the incomplete identification of infective population. Then, we suppose that only a fraction of infectious individuals denoted by ε can be identified by diagnosis.

### 3.3. Multi-Scale Model of COVID-19 Transmission Dynamics

We adapt a previously developed model (1) to describe the transmission dynamics of COVID-19 in Vietnam. The model relies on a microscopic description to capture the movement of the individuals and the transmission of the disease. The model was used to gain insights into the impact of NPIs on the transmission dynamics of COVID-19 in Morocco (2). We study the transmission dynamics of COVID-19 in a population of 250 individual walking randomly in a square domain of 250 ×250 m. This corresponds to a population density of 1,000 inhabitant/km^2^, which the minimal density in urban areas as defined by the U.S. Census Bureau (16, 17). The contact frequency of individuals increases as the population density grows (1, 18). We consider a closed system of individuals and we assume periodic boundary conditions on the movement of individuals to approximate larger systems. The agent-based aspect of the model makes it suitable to study the fine-grained aspects related to the impact of non-pharmaceutical measures. In particular, those which aim to reduce the movement of the population and reduce the chances of disease transmission. A snapshot of numerical simulation using the model is shown in Figure 1B. Details of the model implementation are provided in our previous study (1). In this section, we provide a summary of the main features of the model.

#### 3.3.1. The Movement of Individuals

We use a social force model (19) to describe the movement of each individual agent. The model was used previously to describe the movement of pedestrian in crowded areas (20). We model each agent as a sphere particle which is subjected to several forces. We apply Newton's law to the center of each individual:

(3) $$\begin{array}{l} m_{i}\frac{dv_{i}}{dt}=f_{i}^{self}+\xi_{i} \end{array}$$

(4) $$\begin{array}{l} \frac{dx_{i}}{dt}=v_{i}, \end{array}$$

where *x*_*i*_ is the displacement of the individual, *v*_*i*_ is its velocity, *m*_*i*_ is its mass, ξ_*i*_ is a random perturbation and *f*^*self*^ is the self-driven force defined by:

$$f_{i}^{self}=m_{i}\frac{v_{d,i}-v_{i}}{\tau_{i}},$$

*v*_*d,i*_ represents the desired velocity at which the i-th pedestrian wants to move. We consider that individuals tend to move to random directions, the amplitude of the desired speed is chosen to be following a normal distribution with an average of 1.34 m·s^−1^ (≃5 km/h) and a standard deviation of 0.26 m·s^−1^, τ_*i*_ is a relaxation time.

#### 3.3.2. Modes of Disease Transmission

Infectious individuals can transmit the disease by the mean of secreted droplets that bear the virus. These droplets can be inhaled by neighboring individuals. They also contaminate the neighboring surfaces. We consider both these modes of disease transmission in the model. First, we infectious individuals can infect susceptible individuals if the distance between them is <1 m. Direct transmission depends on the incidence of sneezing, coughing, breathing without mask, or handshaking. Therefore, we assume that the susceptible individual has a probability of *p*_*d*_ to contract the virus upon direct contact with infectious individual. We model the mode of indirect transmission by considering that infectious contaminate neighboring surfaces by secreting droplets which contain the virus. These surfaces can subsequently transmit the virus to susceptible individuals. We describe the concentration of stable SARS-CoV-2 on surfaces as follows:

(5) $$\begin{array}{l} \frac{\partial C}{\partial t}=W-\sigma C, \end{array}$$

where *W* is the secretion rate of the virus by infected individuals and σ is the decay rate of stable SARS-CoV-2. The probability of viral infection by touching surfaces is estimated at:

(6) $$\begin{array}{l} p_{in}=\lambda \bar{C}\left(x_{i}\right), \end{array}$$

where λ is a positive constant taken smaller than one and $\bar{C}\left(x_{i}\right)$ is the normalized concentration of the virus on local contaminated surfaces. Note that we only evaluate the possibility of indirect transmission once each day for each individual at a random moment of the day as the possibility of indirect transmission can be considered as a rare event.

#### 3.3.3. Clinical Course of Infected Patients

Infected individuals do not develop symptoms until the end of the incubation period. However, they start transmitting the virus a day before the onset of symptoms. The median value for the incubation period is 5.1 days and can be sampled using a log-normal distribution (21, 22). After the onset of symptoms, infected individuals get isolated and go into quarantine at home or in a hospital. In this period of quarantine, the patient stops moving in the computational domain and interacting with other individuals. This phase can have two outcomes: the patient can either die or survive. If he or she survives, then they start moving and interacting with other individuals as before, but they become immunized to new infections. Recovered individuals refer to the state of individuals that already contracted the virus and no longer show symptoms. While immunity to re-infection by SARS-CoV-2 is still under investigation (23), we assume recovered individuals to be immune to new SARS-CoV-2 infections in the next few months. Median values for characteristic periods and the distribution used for their sampling them are given in Supplementary Table 2. The clinical evolution of infected individual is represented in Figure 1C.

#### 3.3.4. Demographic Characteristics and Mortality Risks

We assume that the death probability for each patient depends on its characteristics and in particular age and pre-existing risk factors. The considered age-structure is introduced as a distribution function and used to sample the age of each patient. We restrict the population of individuals to the individuals older than 18. This is because individuals who are younger than 18 are less impacted by the disease and most of them are asymptomatic. We consider that the age of the individuals determines the risk of COVID-19-related mortality as represented in Supplementary Table 3 (24).

Furthermore, we consider that individuals can also have one or many of the pre-existing risk factors which increase the mortality risk of COVID-19. These risk factors include chronic respiratory diseases, cardiovascular diseases, elevated blood pressure, diabetes, and cancer. We consider that the prevalence of these health conditions to be similar to the one observed in society. Supplementary Table 4 provides the prevalence of these conditions and the corresponding death probability taken from a World Health Organization (WHO) report (25).

#### 3.3.5. Computational Implementation

We used temporal integration to solve all the equations of the model. We took a very small-time step *dt* = 10^−4^ h = 4.16 ×10^−6^ day to track all contacts between individuals. We use the Euler implicit scheme to solve the Newton's second law of dynamics and the finite different method to solve the equation for SARS-CoV-2 distribution (5). The model is implemented using the C++ language. To ensure code modularity, we have used an object-oriented programming (OOP) architecture. The post-processing of the results was done using the ParaView software and python scripts. The code can be accessed at: https://github.com/MPS7/SIM-CoV.

## 4. Results

### 4.1. The SEIR Model Describes the Impact of NPIs on the Spread of the Disease

We begin by determining the parameters of the SEIR model that describe the evolution of the Covid-19 epidemic in Vietnam. Then we evaluate the impact of different prevention and control strategies on the spread of COVID-19. To achieve this, we use the least square method to fit the parameter β. Then, we reduce this parameter to model the impact of non-pharmaceutical interventions which aim to reduce the contact probability between individuals. Indeed, a lockdown aims to reduce the portion of mobile population which downregulates the contact rate. We estimate the basic reproduction number $R_{0}$ for the epidemic COVID-19 in Vietnam. It is defined as the average number of new infections caused by an infectious individual in a susceptible population. This number describes the propagation speed of the epidemic. It is estimated using the following formula (26):

$$R_{0}=\frac{\beta S_{0}}{\mu_{I}}=\frac{\beta }{\mu_{I}}\left(1-E_{0}-I_{0}-R_{0}\right)=\frac{\beta }{\mu_{I}}\left(1-\frac{X\left(0\right)}{\varepsilon N}\right).$$

In the absence of any prevention or control strategy, the estimated basic reproduction number is $R_{0}=4$. We can see that the value of the basic reproduction number is much higher than the same number at the beginning of the COVID-19 outbreak in Vietnam.

There exist other methods to estimate the basic reproduction number. For comparison purpose, we will use the method presented in a previous study (6). It defines the basic reproduction number as:

$$R_{0}=1+\gamma T_{g}+\rho \left(1-\rho \right)\left(\lambda T_{g}^{2}\right),$$

where *T*_*g*_ is the generation time, ρ denotes the ratio of incubation period to generation time and we define γ as follows:

$$\gamma =\frac{\ln\;Y\left(t\right)}{t}.$$

Here *Y*(*t*) denotes the number of symptomatic cases by time *t*. It can be defined as *Y*(*t*) = *iks*, where *i* is the number of confirmed cases, *s* is the number of susceptible cases, and *k* is the ratio of suspected to confirmed cases, equal taken equal to 0.695 (27). Applying this method to the results of numerical simulations, we estimate $R_{0}=3.34$. Note that this method does not require the value of the contact rate (β) for the calculation of $R_{0}$. As a result, it is possible to calculate the $R_{0}$ using solely graphical data and the generation time of the disease.

**Figure 2:**
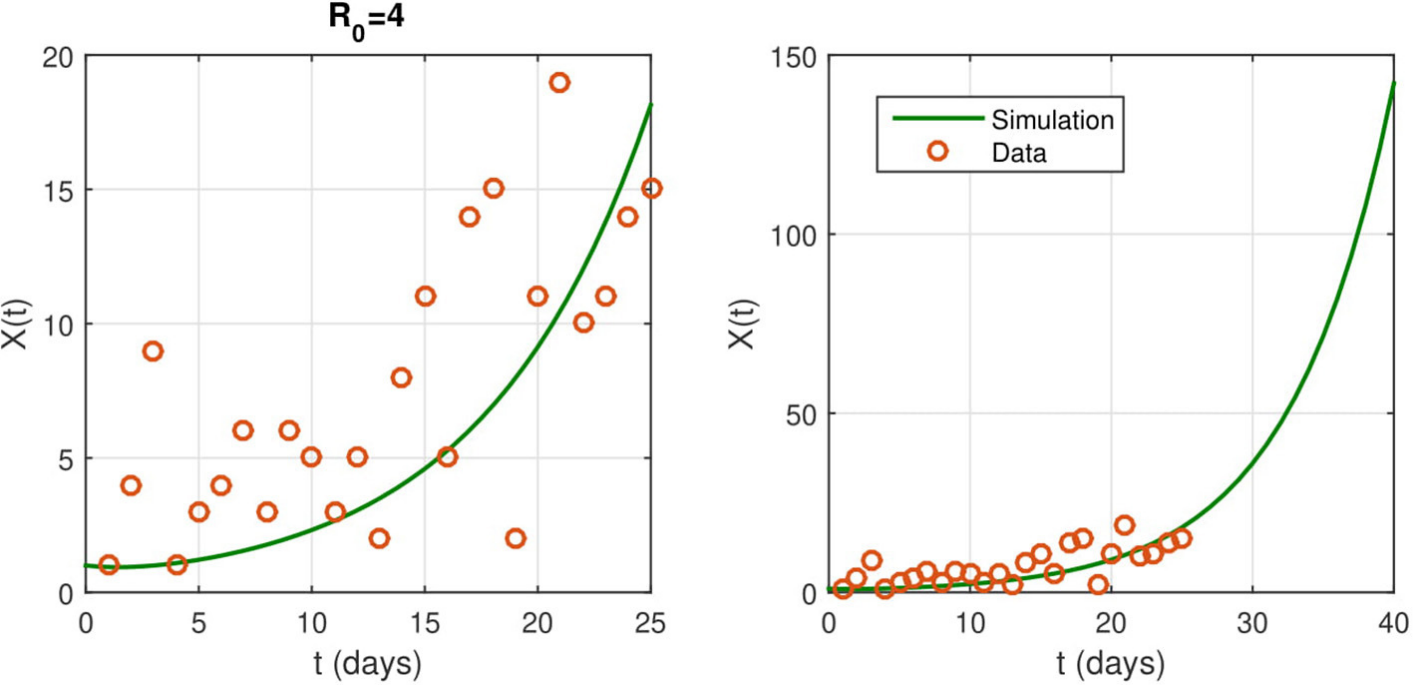
**(Left)** The fitted curve for epidemic evolution, with ε = 0.08. The small red circles are the reported case data. **(Right)** an illustrations of the shape of *X*(*t*) without any intervention. It predicts the spread of the epidemic.

Parameter values for numerical simulation are given in Supplementary Table 1. We estimate the contact rate to be β = 0.4. The identification rate ε does not affect much the basic reproduction number and the rate of infection. In numerical simulations, we set ε = 0.08 and we provide several predictions on the evolution of the epidemic. We show the model predictions for the evolution of the epidemic in the absence of NPIs in Figure 2.

**Figure 3:**
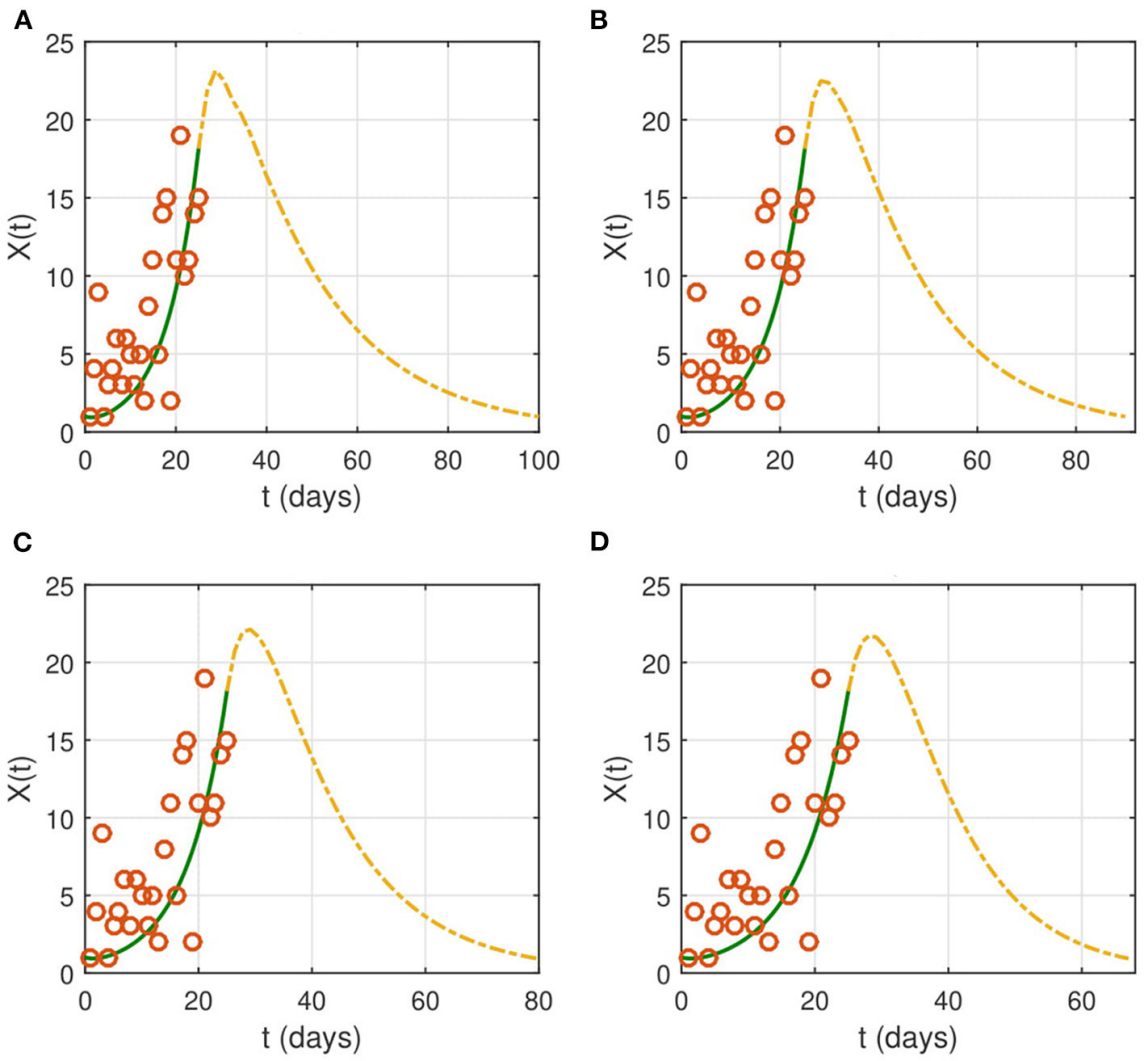
The effect of NPIs on the evolution of the identification function *X*(*t*). Lockdown starts on from April 1, 2020 (*t* = 26). We choose the following values for the contact rate: **(A)** β_*new*_ = 0.1 × β, **(B)** β_*new*_ = 0.08 × β, **(C)** β_*new*_ = 0.05 × β, and **(D)** β_*new*_ = 0.001 × β. The time interval of the X-axis represents the duration of the epidemic. The red circles represent the data for the number of reported cases.

We study the effect of non-pharmaceutical interventions with several cases of severity of the restrictions on the spread of COVID-19. We also explore the influence of the intervention duration. As stated before, the nationwide lockdown started on April 1, 2020. In Figure 3, we reduce the contact rate β to 0.1 × β as soon as the intervention starts. This corresponds to a reduction of contacts by ninety percent of the normal value.

**Figure 4:**
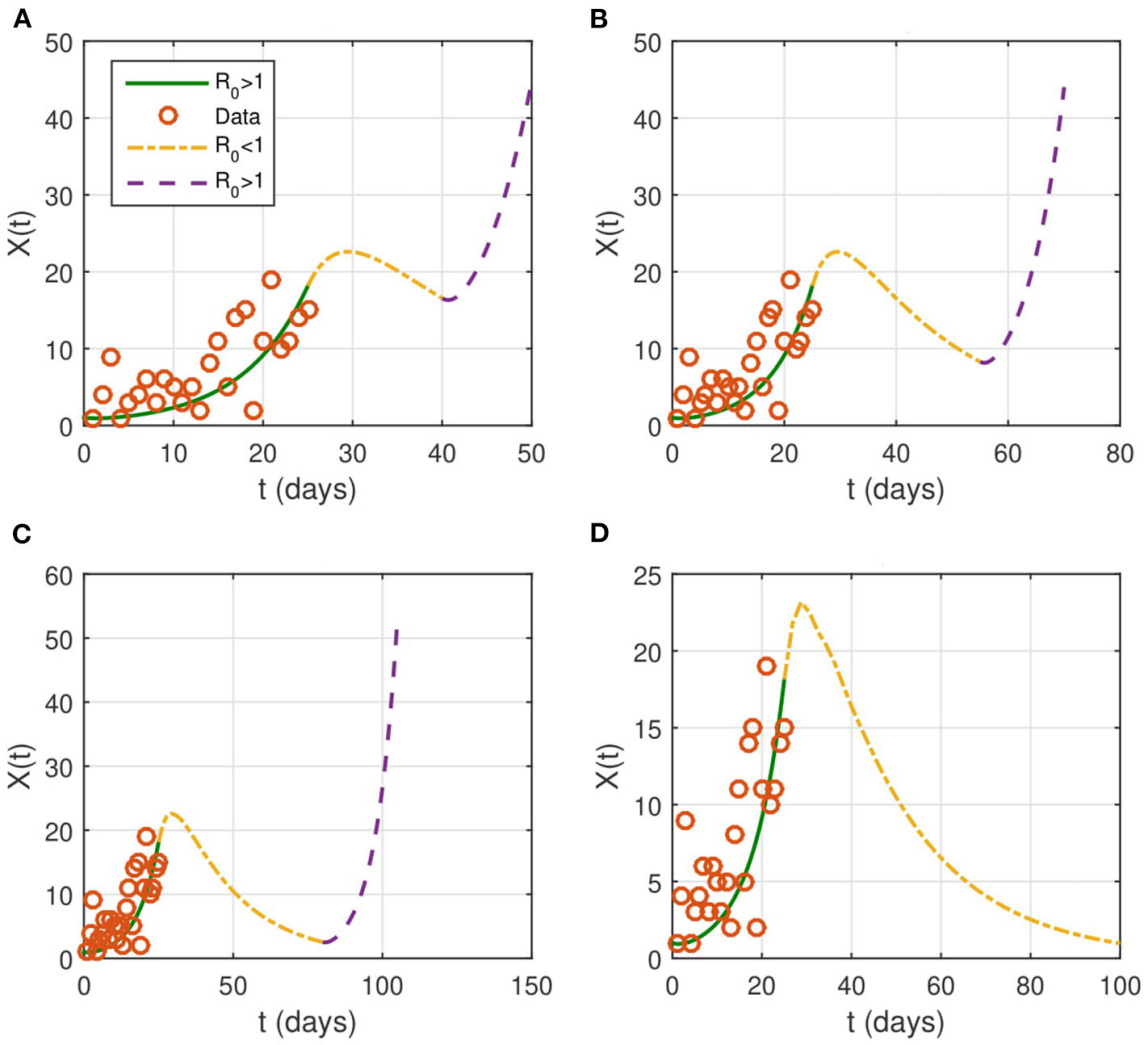
Evolution of the identified infected population of *X*(*t*). NPIs are considered from April 1 (*t* = 26), with β_*new*_ = 0.1 × β and last for a duration *T*. We take several intervention's duration: **(A)** *T* = 15 days, **(B)** *T* = 30 days, **(C)** *T* = 45 days, and **(D)** *T* = 75 days. The red circles represent the data for the number of reported cases.

If the contact rate is below 0.08 × β, then the epidemic is contained in <2 months. More strict social distancing measures which reduces the contact rate by more than 95% would contain the epidemic in a period ranging from a month to a month and a half. We then look for the effect of the duration of the intervention on the evolution of the epidemic (Figure 4). The results suggest that the epidemic can persist and resurge again if the duration is not sufficient.

### 4.2. The Multi-Scale Model Quantifies the Effect of Lockdown on the Propagation of the Disease

**Figure 5:**
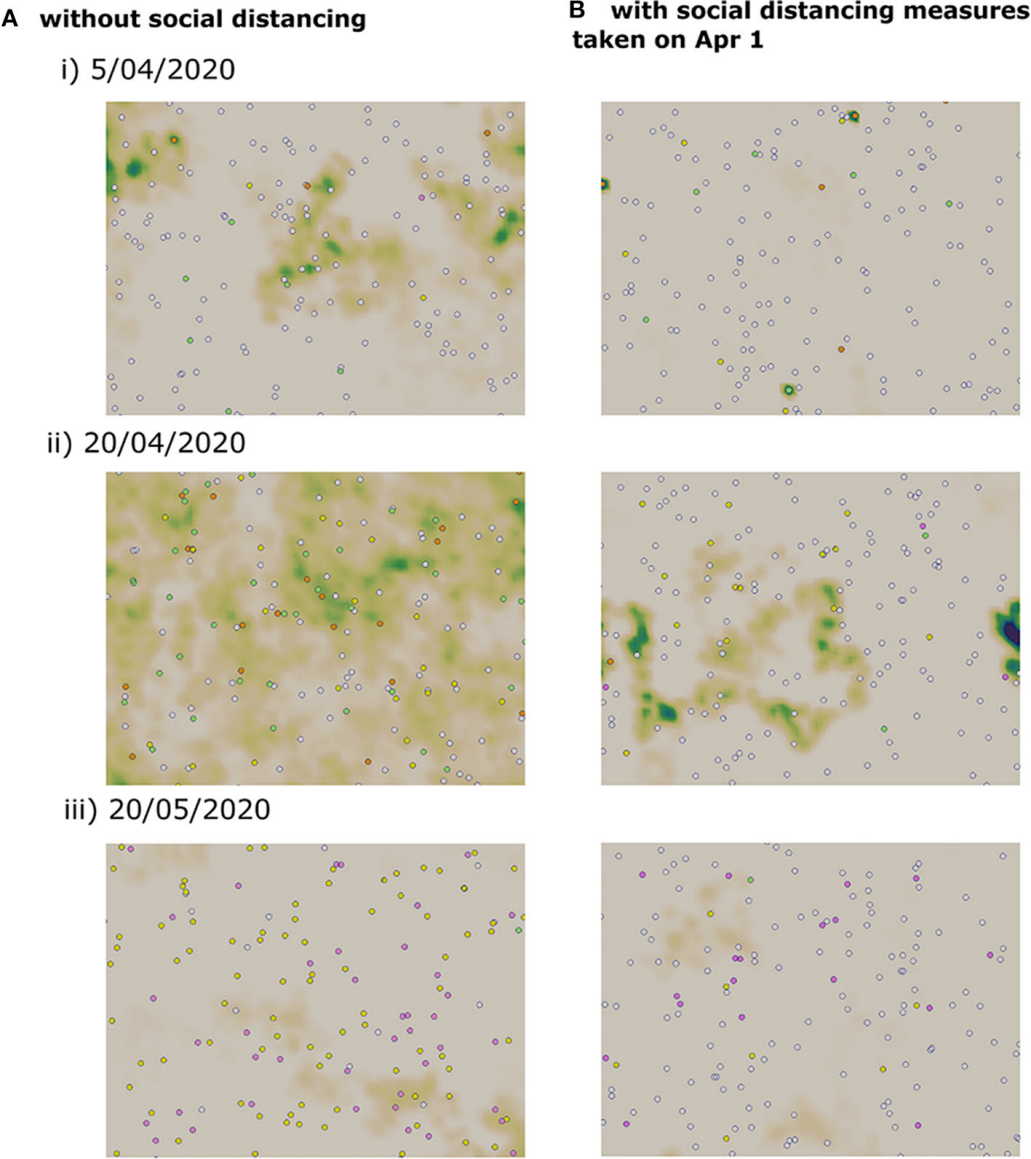
Snapshots of numerical simulations showing the effect of social distancing on the transmission dynamics of COVID-19 in three different dates. **(A)** Without social distancing measures. **(B)** With a partial lockdown which targets 75% of the population.

We estimate the duration of the epidemic to be the difference between the moment of the onset where the number of actively infected individuals exceeds 5% of the population and the time when the same number drops below this value. We compute this duration using the following formula:

$$T_{d}=\left(N_{2}-N_{1}\right)dt,$$

where *N*_2_ is the number of iterations necessary to reach the end of the epidemic, *N*_1_ is the number of iterations to reach the onset of disease spread, and *dt* is the time step. A lockdown is a social distancing measure which aims to reduce the movement of individuals to decrease to the transmission of the disease. However, full lockdown is an intervention that has a relatively high economic and social cost. Therefore, several countries have instead opted for a partial lockdown where only a proportion of the population is immobilized. In Vietnam, a lockdown was mandated on April 1, 2020. In this section, we use the multi-scale model to evaluate the effects of both full lockdown and partial lockdown on the evolution of the epidemic. In this model, we consider that the lockdown corresponds to the case where a portion of the population do not move which reduces the chances of contact between individuals. In the absence of control measures, numerical simulations show that the peak would be reached on May 1, 2020. At this moment, more than half of the population is infected and the virus concentration on the computational domain is high. By the end of the simulation time which corresponds to June 17, 2020, there are still a few cases of infected individuals who are not yet quarantined. Three stages of a numerical simulation showing the transmission dynamics in the absence of control measures are represented in Figure 5A.

We apply a partial lockdown, and we consider that only a half of the population move. In this case, the transmission dynamics remain the same, but the cumulative percent of infected cases is reduced to half. As before, the peak is reached on May 1, 2020, and the active number of infected individuals does not reach zero by the end of the simulation time. When we consider a stricter lockdown and immobilize 75% of the population, the peak is reached on April 18, 2020. The epidemic curve is flattened, and the cumulative percent of infected individuals falls to 29%. Still, the epidemic duration is not shortened and there a few infected cases remain on the computational domain by the end of the simulation time. We have represented three stages of a numerical simulation of the COVID-19 transmission dynamics in Figure 5B.

**Figure 6:**
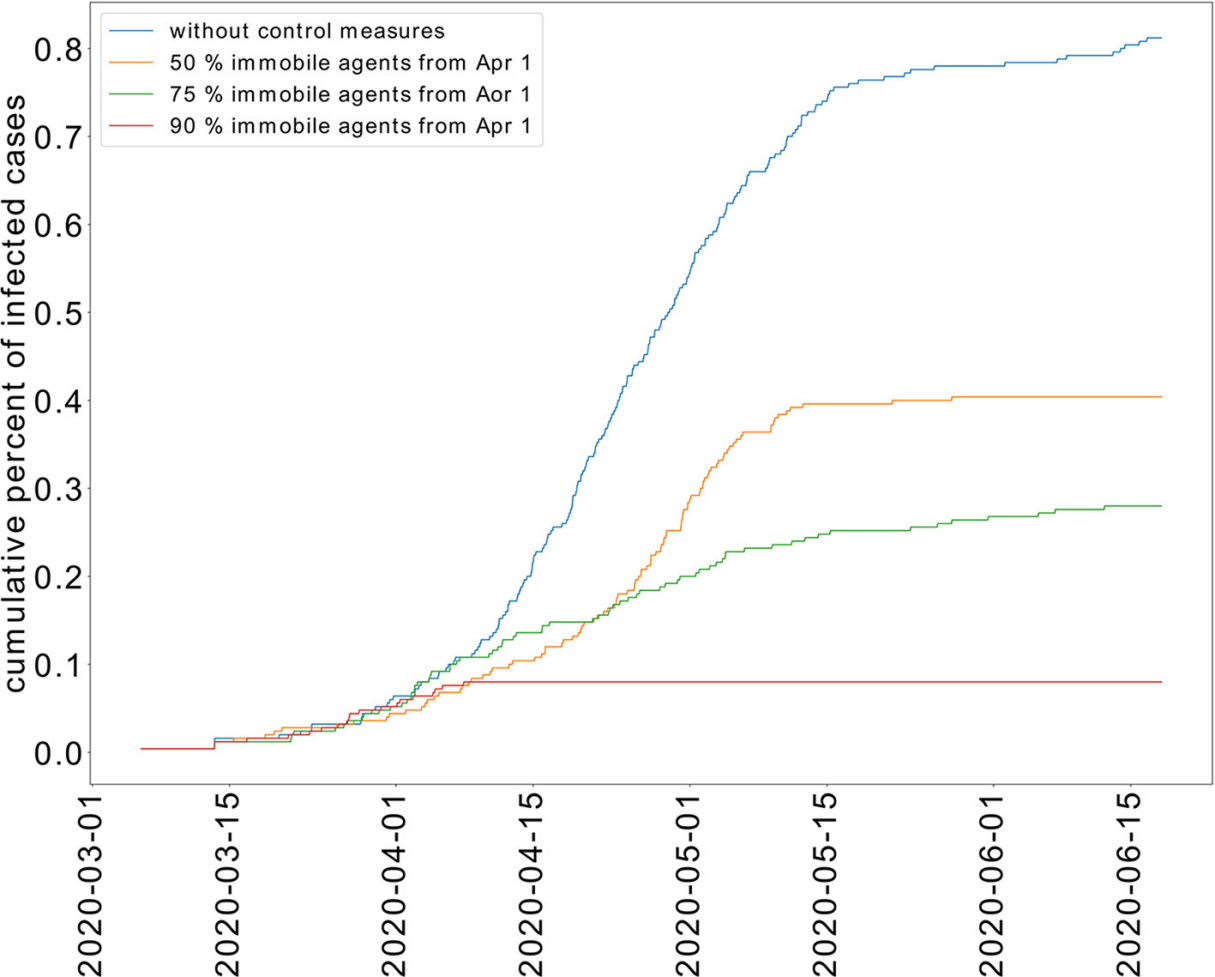
Effect of limited movement of the population on the cumulative percent of infected individuals.

**Figure 7:**
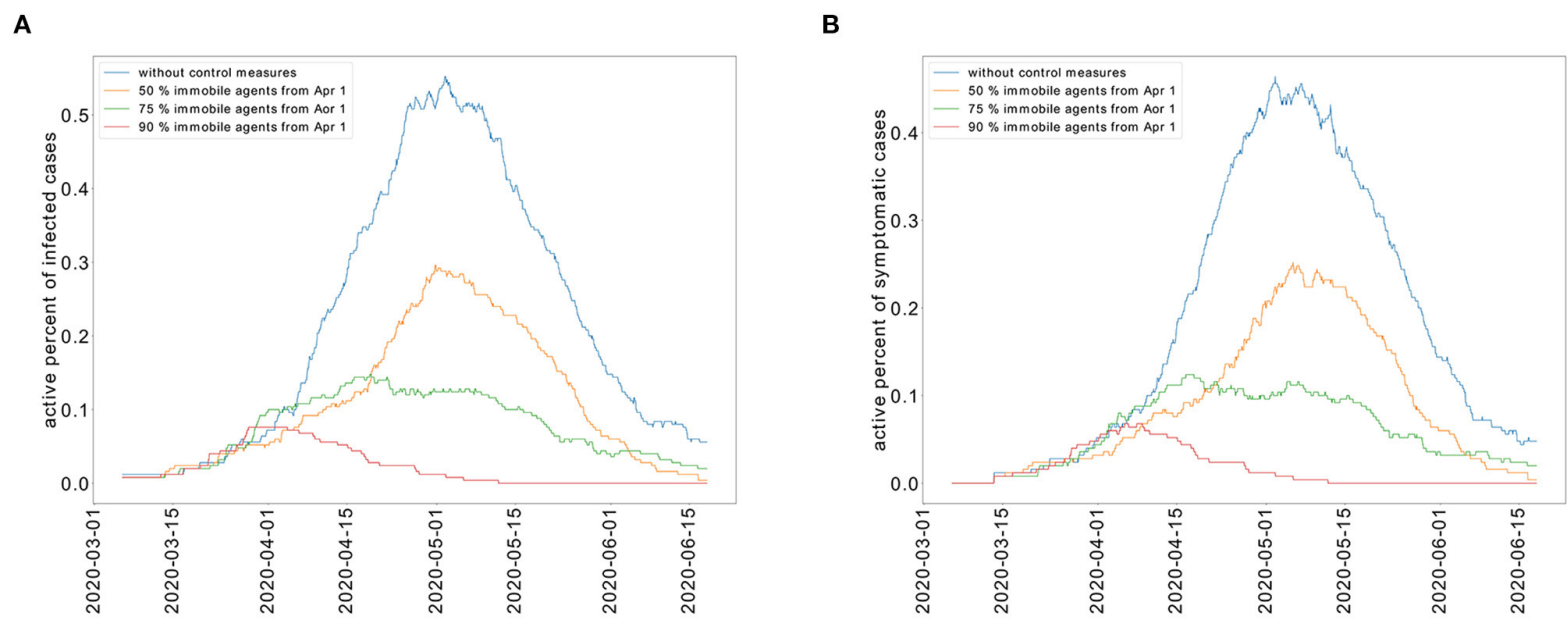
Restricted population movement flattens the epidemic curve for the active percent of infected individuals **(A)** and symptomatic individuals **(B)**.

We study the transmission dynamics when a full lockdown is imposed from April 1, 2020. We consider that 90% of the population cannot move. The adoption of this intervention shortens the time of the epidemic. In this case, the peak is reached on the same day when the lockdown is imposed. The epidemic is resolved by May 15, 2020. The cumulative percent of infected individuals is reduced to 8%. We have represented the cumulative percent of infected individuals over time for the different strategies in Figure 6. The active percent of infected and symptomatic individuals are shown in Figure 7. It shows that a partial lockdown flattens the epidemic curve but does not shorten the duration of the epidemic. Whereas, a full lockdown stops the transmission of the disease and reduces the time of the epidemic.

**Figure 8:**
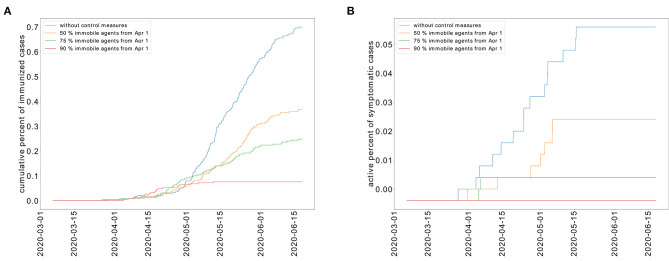
Cumulative percent of recovered individuals **(A)** and deceased patients **(B)** for different levels of restricted movement.

In the absence of control measures, it is expected that 70% of the population would develop immunity against COVID-19. As a result, the population would be less susceptible to a new outbreak. However, this strategy of herd immunity is not guaranteed in the absence of evidence showing that recovered individuals develop immunity to COVID-19. The percent of recovered individuals decreases as the portion of immobilized population increases as shown in Figure 8A. In the absence of control measures, the case fatality rate (CFR) is equal to 6.4%. When a partial lockdown is applied and 50% of the population is immobilized, the CFR is 5.9%. When a full lockdown is imposed, the number of deceased patients reaches zero. We have represented the cumulative percent of deceased individuals for the different epidemic control strategies in Figure 8B. We represent the cumulative number of deceased patients in Figure 8.

## 5. Discussion

This work aims to predict the evolution of the COVID-19 epidemic in Vietnam. To achieve this, we have used the available data on the early stages of the epidemic to calibrate two models. The first is an SEIR model which describes the transmission dynamics using four compartments: susceptible, exposed, infectious, and recovered. The second is a multi-scale model which describes the explicitly the movement of individuals, the transmission of the disease, and the clinical course of patients. The first approach is suitable for the study of the transmission dynamics of the disease at the population level. While the second is appropriate for the investigation of the fine-grained aspects that determine the spread of the disease at the individual level. Therefore, the two approaches complement each other and provide a deeper understanding of the transmission dynamics of COVID-19. We have previously applied these models to study COVID-19 transmission dynamics in Algeria (28), China, Italy (1), and Morocco (2).

Two models are used to predict the effects of a partial and full lockdown on the evolution of the epidemic in Vietnam. Both models show that a partial lockdown would reduce the cumulative number of infected individuals. However, it will take more than 2 months and a half after the lockdown beginning for the epidemic to resolve. Whereas, it takes only 1 month and a half for the epidemic to end the epidemic if a full lockdown is mandated. This last strategy was adopted in Vietnam and it shows the good agreement between the predictions of the two models and reported data as of May 3, 2020.

The two models rely on several assumptions. First, we consider closed systems in both models because international flights are suspended. Second, we do not consider the changes in the population related to the birth of new individuals and deaths by other causes than COVID-19. Third, data related to COVID-19 mortality and infection dynamics are taken from clinical studies conducted in China. Finally, the effects of asymptomatic individuals and unreported cases are not taken into consideration because we do not have sufficient data on the transmission and infection dynamics of these individuals. In a forthcoming work, we plan to apply the model to investigate the transmission dynamics of COVID-19 in urban areas with realistic geometries (13).

## Data Availability Statement

The original contributions generated for the study are included in the article/Supplementary Material, further inquiries can be directed to the corresponding author.

## Author Contributions

All authors designed the study and planned the simulations. AC analyzed the data and conducted numerical simulations with the SEIR model. AB and AJ performed simulations with the multi-scale models. All authors contributed to the writing of the paper and approved it for publication.

## Conflict of Interest

The authors declare that the research was conducted in the absence of any commercial or financial relationships that could be construed as a potential conflict of interest.

## References

[1] Bouchnita A Jebrane A. A hybrid multi-scale model of COVID-19 transmission dynamics to assess the potential of non-pharmaceutical interventions. Chaos Solit Fract. (2020) 138:109941. 10.1016/j.chaos.2020.109941 32834575 PMC7269965

[2] Bouchnita A Jebrane A A multi-scale model quantifies the impact of limited movement of the population and mandatory wearing of face masks in containing the COVID-19 epidemic in Morocco. Math Model Nat Phenom. (2020) 15:13 10.1051/mmnp/2020016

[3] Jia J Ding J Liu S Liao G Li J Duan B Modeling the control of COVID-19: impact of policy interventions and meteorological factors. arXiv. (2020) 200302985.

[4] Volpert V Banerjee M Petrovskii S On a quarantine model of coronavirus infection and data analysis. Math Model Nat Phenom. (2020) 15:24 10.1051/mmnp/2020006

[5] Kuniya T. Prediction of the epidemic peak of coronavirus disease in Japan, 2020. J Clin Med. (2020) 9:789. 10.3390/jcm9030789 32183172 PMC7141223

[6] Fang Y Nie Y Penny M. Transmission dynamics of the COVID-19 outbreak and effectiveness of government interventions: a data-driven analysis. J Med Virol. (2020) 92:645–59. 10.1002/jmv.25750 32141624 PMC7228381

[7] Liu Y Gayle AA Wilder-Smith A Rocklöv J. The reproductive number of COVID-19 is higher compared to SARS coronavirus. J Travel Med. (2020) 27:taaa021. 10.1093/jtm/taaa021 32052846 PMC7074654

[8] Rocklöv J Sjödin H Wilder-Smith A. COVID-19 outbreak on the Diamond Princess cruise ship: estimating the epidemic potential and effectiveness of public health countermeasures. J Travel Med. (2020) 27:taaa030. 10.1093/jtm/taaa030 32109273 PMC7107563

[9] Ajelli M Gonçalves B Balcan D Colizza V Hu H Ramasco JJ. Comparing large-scale computational approaches to epidemic modeling: agent-based versus structured metapopulation models. BMC Infect Dis. (2010) 10:190. 10.1186/1471-2334-10-190 20587041 PMC2914769

[10] Dong W Heller K Pentland AS Modeling infection with multi-agent dynamics. In: *International Conference on Social Computing, Behavioral-Cultural Modeling, and Prediction* Berlin; Heidelberg: Springer (2012). p. 172–9. 10.1007/978-3-642-29047-3_21

[11] Roche B Guégan JF Bousquet F. Multi-agent systems in epidemiology: a first step for computational biology in the study of vector-borne disease transmission. BMC Bioinformatics. (2008) 9:435. 10.1186/1471-2105-9-435 18922166 PMC2600827

[12] Ferguson NM Cummings DA Fraser C Cajka JC Cooley PC Burke DS. Strategies for mitigating an influenza pandemic. Nature. (2006) 442:448–52. 10.1038/nature04795 16642006 PMC7095311

[13] Gomez J Prieto J Leon E Rodriguez A INFEKTA: a general agent-based model for transmission of infectious diseases: studying the COVID-19 propagation in Bogotá-Colombia. medRxiv. (2020). 10.1101/2020.04.06.20056119 PMC7894857 33606714

[14] Garira W. A complete categorization of multiscale models of infectious disease systems. J Biol Dyn. (2017) 11:378–435. 10.1080/17513758.2017.1367849 28849734

[15] Ministry of Health Covid-19 Disease. (2020). Available online at: https://ncov.moh.gov.vn/

[16] US Bureau of the Census Geographic Areas Reference Manual. US Department of Commerce, Economics and Statistics Administration, Bureau of the Census (1994).

[17] Bento AM Cropper ML Mobarak AM Vinha K The effects of urban spatial structure on travel demand in the United States. Rev Econ Stat. (2005) 87:466–78. 10.1162/0034653054638292

[18] Rodríguez A Gómez J Diaconescu A Foraging-inspired self-organisation for terrain exploration with failure-prone agents. In: *2015 IEEE 9th International Conference on Self-Adaptive and Self-Organizing Systems*. Massachusetts, MA: IEEE (2015). p. 121–30.

[19] Helbing D Molnar P. Social force model for pedestrian dynamics. Phys Rev E. (1995) 51:4282. 10.1103/PhysRevE.51.4282 9963139

[20] Kabalan B Argoul P Jebrane A Cumunel G Erlicher S A crowd movement model for pedestrian flow through bottlenecks. Ann Solid Struct Mech. (2016) 8:1–15. 10.1007/s12356-016-0044-3

[21] Linton NM Kobayashi T Yang Y Hayashi K Akhmetzhanov AR Jung SM. Incubation period and other epidemiological characteristics of 2019 novel coronavirus infections with right truncation: a statistical analysis of publicly available case data. J Clin Med. (2020) 9:538. 10.3390/jcm9020538 32079150 PMC7074197

[22] Lauer SA Grantz KH Bi Q Jones FK Zheng Q Meredith HR. The incubation period of coronavirus disease 2019 (COVID-19) from publicly reported confirmed cases: estimation and application. Ann Intern Med. (2020) 172:577–82. 10.7326/M20-0504 32150748 PMC7081172

[23] Ota M Will we see protection or reinfection in COVID-19? Nat Rev Immunol. (2020) 20:351 10.1038/s41577-020-0316-3 PMC7186928 32303697

[24] Surveillances V. The epidemiological characteristics of an outbreak of 2019 novel coronavirus diseases (COVID-19)—China, 2020. China CDC Weekly. (2020) 2:113–22. 10.46234/ccdcw2020.032 34594836 PMC8392929

[25] World Health Organization Coronavirus Disease (COVID-2019) Situation Report Number 41. Geneva: World Health Organization (2020).

[26] Van den Driessche P Watmough J. Reproduction numbers and sub-threshold endemic equilibria for compartmental models of disease transmission. Math Biosci. (2002) 180:29–48. 10.1016/S0025-5564(02)00108-6 12387915

[27] Huang C Wang Y Li X Ren L Zhao J Hu Y. Clinical features of patients infected with 2019 novel coronavirus in Wuhan, China. Lancet. (2020) 395:497–506. 10.1016/S0140-6736(20)30183-5 31986264 PMC7159299

[28] Bentout S Chekroun A Kuniya T. Parameter estimation and prediction for coronavirus disease outbreak 2019 (COVID-19) in Algeria. AIMS Public Health. (2020) 7:306. 10.3934/publichealth.2020026 32617358 PMC7327392

