# Supplementary material for: Mathematical Modeling Predicts That Strict Social Distancing Measures Would Be Needed to Shorten the Duration of Waves of COVID-19 Infections in Vietnam
Source: Front Public Health. 2021 Jan 12;8:559693. doi: 10.3389/fpubh.2020.559693 (PMC7841962; doi:10.3389/fpubh.2020.559693)
Supplement: Supplementary file 1 [file Data_Sheet_1.PDF]

Supplementary Material: Mathematical modelling predicts that strict social distancing measures would be needed to shorten the duration of the COVID-19 epidemic in Vietnam

1 NUMERICAL VALUES FOR THE PARAMETERS OF THE SEIR MODEL

We present the numerical values for the parameters used in the SEIR in Table S1 as well as the references from where they were taken.

Parameters	Description	Value	Reference
β	Contact rate	0.4	Estimated
μ_I	Removal rate	0.1	(1, 7)
μ_E	Onset rate	0.2	(1, 4, 6)
$\frac{1}{\mu_I}$	The average infectious period	10	(1, 7)
$\frac{1}{\mu_E}$	The average incubation period	$1/5$	(1, 4, 6)
ε	Identification rate	0.01-0.1	(2)
N	Total population in Vietnam	$97,338,579$	

Table S1. Parameter values for numerical simulations with SEIR model.

2 NUMERICAL VALUES FOR THE PARAMETERS OF THE MULTI-SCALE MODEL

In this section, the numerical values for the parameters used in the multi-scale model are presented. Table S2 provides the values for parameters for the epidemiological characteristics of the disease, Table S3 for the age distribution of agents and the mortality of each age group, and Table S4 for the prevalence of the COVID-19 mortality risk factors and the death probability of each of them.

characteristic time	median (standard deviation)	distribution and reference
Incubation period	5.1 (3.1) days	log-normal distribution (3)
Pre-symtomatic transmission	1 day	assumed
Onset to quarantine	3.9 (0.5) days	normal distribution (5)
Quarantine to discharge	20.2 (2) days	normal distribution (5)
Quarantine to death	13 (1) days	normal distribution (5)

Table S2. Values of the COVID-19 epidemiological characteristics in the model.

Age group	Sampling probability in the model	Death probability
18-29	19.24 %	0.2 %
30-39	19.24 %	0.2 %
40-49	19.24 %	0.4 %
50-59	19.24 %	1.3 %
60-69	14.57 %	3.6 %
70-79	6.08 %	8 %
80-90	2.39 %	14.8 %

Table S3. The prevalence of age groups in the model and the corresponding death probabilities for each group.

Risk factor	Sampling probability in the model	Death probability
Cardiovascular diseases	25 %	13.2 %
Diabetes	8 %	9.2 %
High blood pressure	25 %	8.4 %
Chronic respiratory diseases	5 %	8 %
Cancer	0.57 %	7.6 %

Table S4. The prevalence of COVID-19 mortality risk factors in the model and the corresponding death probability.

REFERENCES

1 .Anderson, R. M., Heesterbeek, H., Klinkenberg, D., and Hollingsworth, T. D. (2020). How will country-based mitigation measures influence the course of the covid-19 epidemic? *The Lancet* 395, 931–934

2 .Kuniya, T. (2020). Prediction of the epidemic peak of coronavirus disease in japan, 2020. *Journal of Clinical Medicine* 9, 789

3 .Lauer, S. A., Grantz, K. H., Bi, Q., Jones, F. K., Zheng, Q., Meredith, H. R., et al. (2020). The incubation period of coronavirus disease 2019 (covid-19) from publicly reported confirmed cases: estimation and application. *Annals of internal medicine*

4 .Li, Q., Guan, X., Wu, P., Wang, X., Zhou, L., Tong, Y., et al. (2020). Early transmission dynamics in wuhan, china, of novel coronavirus–infected pneumonia. *New England Journal of Medicine*

5 .Linton, N. M., Kobayashi, T., Yang, Y., Hayashi, K., Akhmetzhanov, A. R., Jung, S.-m., et al. (2020). Incubation period and other epidemiological characteristics of 2019 novel coronavirus infections with right truncation: a statistical analysis of publicly available case data. *Journal of clinical medicine* 9, 538

6 .[Dataset] Organization, W. H. et al. (2020). Coronavirus disease (covid-2019) situation report number 41

7 .Zou, L., Ruan, F., Huang, M., Liang, L., Huang, H., Hong, Z., et al. (2020). Sars-cov-2 viral load in upper respiratory specimens of infected patients. *New England Journal of Medicine* 382, 1177–1179
